# Artificial Intelligence in Public Health: Revolutionizing Epidemiological Surveillance for Pandemic Preparedness and Equitable Vaccine Access

**DOI:** 10.3390/vaccines11071154

**Published:** 2023-06-26

**Authors:** Pranav Anjaria, Varun Asediya, Prakrutik Bhavsar, Abhishek Pathak, Dhruv Desai, Veerupaxagouda Patil

**Affiliations:** 1 College of Veterinary Science and Animal Husbandry, Kamdhenu University, Anand 388001, Gujarat, India; 2 Apollo College of Veterinary Medicine, Jaipur 302031, Rajasthan, India; 3 Department of Pathobiology, School of Veterinary Medicine, University of Pennsylvania, Philadelphia, PA 19104, USA; 4 Fischell Department of Bioengineering, A. James Clark School of Engineering, University of Maryland, College Park, MD 20742, USA

Epidemiological surveillance involves systematic gathering, analysis, interpretation, and sharing of health data, with the goal of preventing and controlling diseases. Artificial Intelligence (AI)-based epidemiological surveillance is a promising approach to detecting, monitoring, and predicting the spread of diseases that employs AI technologies to analyze data from multiple sources, such as electronic health records, social media, and news articles [1]. By identifying real-time trends, these systems provide relevant insights to health officials, enabling disease outbreak responses that effectively protect public health. AI offers a significant advantage over traditional disease surveillance methods due to its ability to predict future outbreaks, empowering public health officials to take proactive and preventive measures at an early stage. Moreover, AI-based systems dynamically learn from new data, continuously improving their predictive accuracy [2], thereby enhancing the effectiveness of disease surveillance. This adaptive learning capability means that AI systems are superior to traditional methods, which are more static and lack the sensitivity required to accurately forecast outbreaks and identify emerging diseases.

“Arogya Setu” was developed by the Indian Government in collaboration with public and private stakeholders, including the National Informatics Centre (NIC) under the Ministry of Electronics and Information Technology, to deliver real-time contact tracing and self-assessment information to individuals, helping them to remain informed during critical periods, such as the COVID-19 pandemic. Though the app has only been downloaded 210 million times in India, its massive user base allowed extensive data collection and analysis, enabling the AI system to provide accurate insights and recommendations to individuals and health authorities about how to best combat the COVID-19 pandemic. Its AI-driven self-assessment tools and contact tracing features helped to contain the spread of the virus and saved countless lives across India. “BlueDot” is another notable AI-powered platform that was developed by epidemiology and data science experts in Canada, using machine learning algorithms to analyze various data sources, including news reports, airline ticketing data, and other indicators [3]. This AI-based driven software provides insights about diseases attributes, case counts, healthcare capacity analyses, epidemic preparedness, environmental suitability, and risk alert warming. For example, BlueDot provided early warnings to public health officials regarding the emergence of COVID-19 in Wuhan, China, in late 2019, thus influencing the global response to the pandemic. “HealthMap” is another AI-based epidemiological information surveillance system, evolving from the Children’s Hospital Informatics Program (CHIP) based in Boston, Massachusetts, USA. It monitors, organizes, and visualizes disease outbreaks based on geographical location, time, and infectious pathogens by analyzing data from varied sources, including social media, news reports, and official health reports. It can detect the spread of COVID-19 in real time [4], allowing public health officials to track the virus’ spread and launch targeted interventions. Moreover, the Centers for Disease Control and Prevention (CDC) in the United States adopted AI-based epidemiological surveillance to monitor the spread of infectious diseases. Their system, known as “BioSense”, uses machine learning algorithms to analyze data from electronic health records, emergency department visits, and other sources to identify infectious diseases outbreaks [5]. Thus, AI- based software utilizes various data sources as represented in Figure 1 for various techniques to analyze, process, and issue early alerts as mentioned in Figure 2.

AI-based technological advances can also minimize pandemic-related disparities and broaden access to medical care. For example, AI-based epidemiological surveillance can ensure more equitable vaccine access. The World Health Organization (WHO), the International Monetary Fund (IMF), and other leading international organizations observed vaccine-access inequality during the COVID-19 pandemic. Though initially a production- and international supply chain-related issue, following national vaccine roll-outs, vaccine-access inequality was primarily caused by unequal in-country vaccine distribution and vaccine hesitancy [6]. In turn, the lack of data, verified information, health records, education, and digitalized surveillance systems caused problems related to unequal vaccine uptake. AI-powered systems can streamline vaccine production, supply chain management, distribution channels, educational materials, and data collection based on the geographical and demographic characteristics of target populations, ensuring that vaccines can equitably reach all individuals. Thus, adoption of AI-based systems can reduce infection rates, vaccine hesitancy, and vaccine inequality.

One potential downside is the risk of software generating false positive or false negative test results. AI-based systems may identify patterns or trends unrelated to disease outbreaks or miss important signals due to limitations in the algorithms or available data. This limitation underscores the need for ongoing monitoring and evaluation to ensure the lasting effectiveness of AI-based epidemiological surveillance. Another challenge is protecting personal data against data privacy- or security-related problems. For example, AI systems may collect and analyze sensitive data, such as personal health information or social media activity, which must be securely stored, protected, and used. Public trust in these systems may be compromised if individuals feel that organizations fail to respect their right to data privacy. Proper regulation and oversight of AI-based epidemiological surveillance systems is also required to guarantee their responsible and ethical use. Clear data collection, sharing, and analyses regulations, as well as risk mitigation strategies, are required to maintain public trust.

AI-based health surveillance systems have several important public health policy implications. Firstly, it is crucial to establish regulations and standards for data privacy and security to protect sensitive data acquired from multiple sources. Secondly, clear guidelines are required to prevent the misuse of AI-based surveillance systems. Thirdly, investment in research and development is also necessary to improve the accuracy and effectiveness of these systems; innovation should prioritize refining algorithms and integrating emerging data sources. Fourthly, investment in data science and ethics training for public health officials can ensure responsible use of these systems. Finally, the ethical and social implications of AI-based epidemiological surveillance need to be addressed, including issues of bias and discrimination in algorithmic decision-making, to ensure that vulnerable populations are not unfairly targeted or stigmatized. These policy implications are complex and multifaceted, with collaboration between policymakers, public health officials, and other stakeholders required to mitigate potential problems.

Future directions for AI-based epidemiological surveillance-related research include developing refined algorithms that can handle complex data sets, thus improving the accuracy of disease detection and prediction; improving individualized records of vaccine provision and outreach; and gathering more comprehensive insights regarding the health statuses of different populations. Another area of focus is the development of real-time surveillance systems to detect disease outbreaks and provide early warnings to public health officials. Finally, efforts to ensure data privacy and security will be crucial to maintaining public trust and acceptance of AI-driven medical technologies.

## Figures and Tables

**Figure 1:**
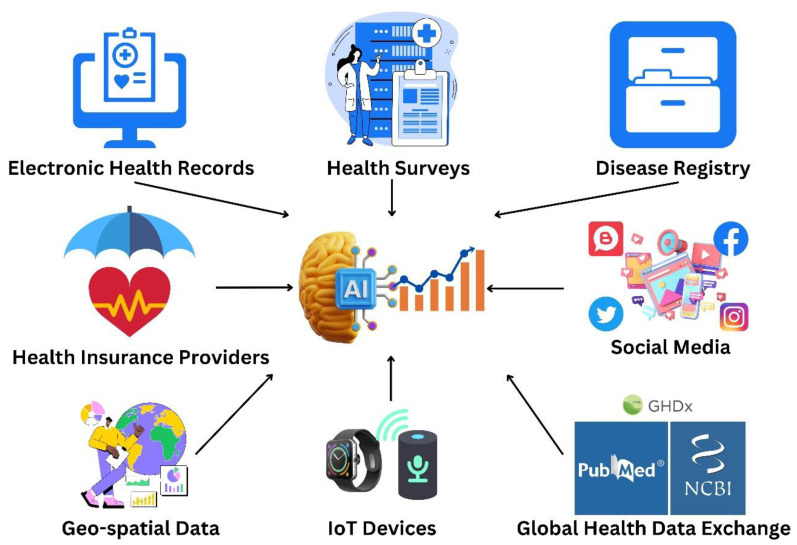
Diverse sources that provide data to Artificial Intelligence-powered health systems.

**Figure 2:**
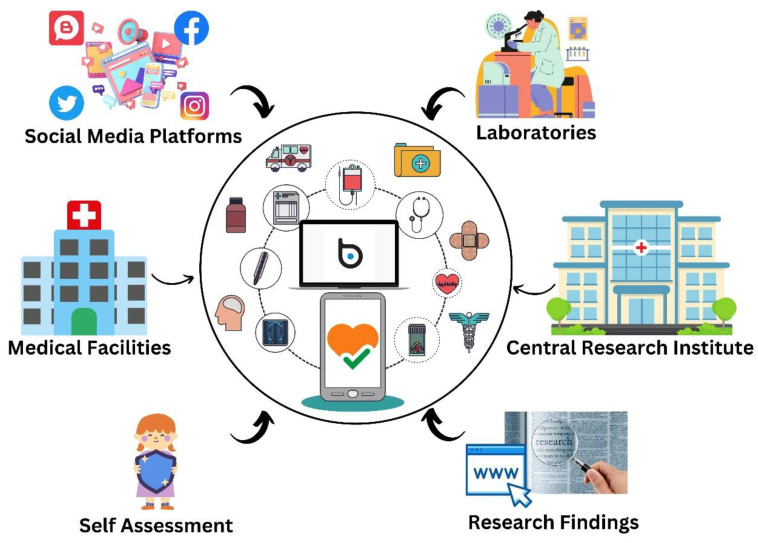
Techniques used by AI-based healthcare software to issue early alerts and improve public health.

## Data Availability

All data are represented.
